# Efficiency of Monocyte/High-Density Lipoprotein Cholesterol Ratio Combined With Neutrophil/Lymphocyte Ratio in Predicting 28-Day Mortality in Patients With Sepsis

**DOI:** 10.3389/fmed.2021.741015

**Published:** 2021-10-13

**Authors:** Jing-yan Li, Ren-qi Yao, Shuang-qing Liu, Yun-fei Zhang, Yong-ming Yao, Ying-ping Tian

**Affiliations:** ^1^Department of Emergency, The Second Hospital of Hebei Medical University, Shijiazhuang, China; ^2^Translational Medicine Research Center, Medical Innovation Research Division and Fourth Medical Center of the Chinese PLA General Hospital, Beijing, China

**Keywords:** neutrophil/lymphocyte ratio, monocyte/high-density lipoprotein cholesterol ratio, predictive value, mortality, sepsis

## Abstract

**Background:** Sepsis can cause unpredictable harm, and early identification of risk for mortality may be conducive to clinical diagnosis. The present study proposes to assess the efficacy of the monocyte/high-density lipoprotein cholesterol ratio (MHR) combined with the neutrophil/lymphocyte ratio (NLR) on the day of admission in predictive efficacy in the 28-day mortality risk in critical patients with sepsis.

**Material and Methods:** We administered observational and retrospective cohort research from a single center. The correlation of the clinical variables, together with the system severity scores of APACHE II and SOFA, are displayed by correlation analysis, and a Cox regression model could be performed to screen the independent risk factors and estimate the capacity of multiple markers in predicting 28-day mortality. The receiver operating characteristic (ROC) curve served as an applied method to output cutoff values for the diagnosis and prognostic risk, and the area under the ROC curve and net reclassification improvement index (NRI), as well as integrated discrimination improvement index (IDI) were employed to assess the feasibility of multiple parameters for predictive value in 28-day mortality of septic patients.

**Results:** The study enrolled 274 eligible patients with sepsis. The correlation analysis indicated NLR and MHR were related to the sepsis severity. A multivariate Cox regression analysis indicated that NLR together with MHR displayed a close relation to death rate after adjusting for other potential confounders (NLR, HR = 1.404 [95% CI 1.170–1.684], *P* < 0.001; MHR, HR = 1.217 [95% CI 1.112–1.331], *P* < 0.001). The AUC of NLR, MHR, NLR_MHR was 0.827, 0.876, and 0.934, respectively. The addition on the biomarker NLR_MHR to the prediction model improved IDI by 18.5% and NRI by 37.8%.

**Conclusions:** Our findings suggest that NLR and MHR trend to an elevated level in non-surviving patients with sepsis. Evaluation of NLR_MHR, an independent risk factor for increased mortality, might improve the predictive efficacy for 28-day mortality risk in septic patients.

## Introduction

Sepsis is a complicated, life-threatening disorder attributed to a dysregulated host response to infection; eventually acute multiorgan dysfunction develops with high morbidity and mortality (1–3). This syndrome, one of the major causes of death in the intensive care unit (ICU), is universally accepted as a public health issue with a considerable economic burden and tremendous concern for critical patients (4–6). According to the reports from the Centers for Disease Control, the incidence of sepsis is approximately more than 750,000 cases per year globally, and the morbidity of sepsis in all ICU admissions is as high as 27% (7, 8). Because of sepsis being the final pathway to death from most infections, it remains at a high mortality at around 25–30% in hospitalized sepsis patients that are equivalent to killing tens of millions of individuals worldwide annually (9–11). Despite constant progress in patient administration and therapeutic strategies, sepsis remains an intractable problem in clinical care because of the limitations in the gold standard of sepsis diagnosis as well as timely identification, which hinders the implement by reference in epidemiological studies.

The international consensus definition for sepsis and septic shock (Sepsis 3.0) has redefined sepsis as fatal multiple organ dysfunction with systemic interaction between excessive inflammatory response and a suppressive immune state in response to an infectious organism or tissue injury (12). The consensus of Sepsis 3.0 emphasizes the immune system as the foundation at which host-derived molecules and foreign products induced by pathogenic microorganism interact with pathogen recognition receptors expressed on immune cells, which cause unbalanced activation of innate immunity (13). Moreover, it emphasizes that the interactions between systemic inflammation and oxidative stress have particularly been accused of performing crucial impact on the pathogenesis of sepsis (14–16). Increasing evidence supports the viewpoint that both immune dysfunction and oxidative stress are critical in the pathogenesis of sepsis. Along with the continual amplification of immune dissonance, oxidative stress is exacerbated during sepsis, finally leading to the redox cascade of cell damage, impairment of mitochondrial function, and aggravation of inflammation (17). Thus, septic patients at risk of immune deterioration and oxidative storm should be identified prior to the onset of organ dysfunction. Identifications of immune and oxidative-related predictors in sepsis have great potential to improve the diagnosis, assessment, and treatment of septic complications.

Although various biomarkers have been improved and applied in evaluation of the capacity of early recognition and prediction in sepsis (18–20), their exact values are still uncertain or controversial. The neutrophil/lymphocyte ratio (NLR) is a rapidly available parameter that is previously reported as reflecting the severity of the disease in critically ill patients and notably correlates with in-hospital mortality in sepsis (21). However, whether NLR predicts septic prognosis in the long term remains controversial, and the reason is that NLR is representative only of the quantity of immune cells rather than functional and oxidative status when sepsis occurs. Monocyte/high-density lipoprotein cholesterol ratio (MHR) is proven to be a parameter of systemic inflammation and oxidative stress in many inflammatory diseases (22–24). Hence, it is speculated that MHR together with NLR might further improve predicting mortality risk in septic patients better than a single indicator. In light of this evidence, we propose to investigate the effectiveness of MHR combined with NLR in predicting 28-day mortality in patients with sepsis.

## Materials and Methods

### Subject Design and Patient Enrollment

**Figure 1:**
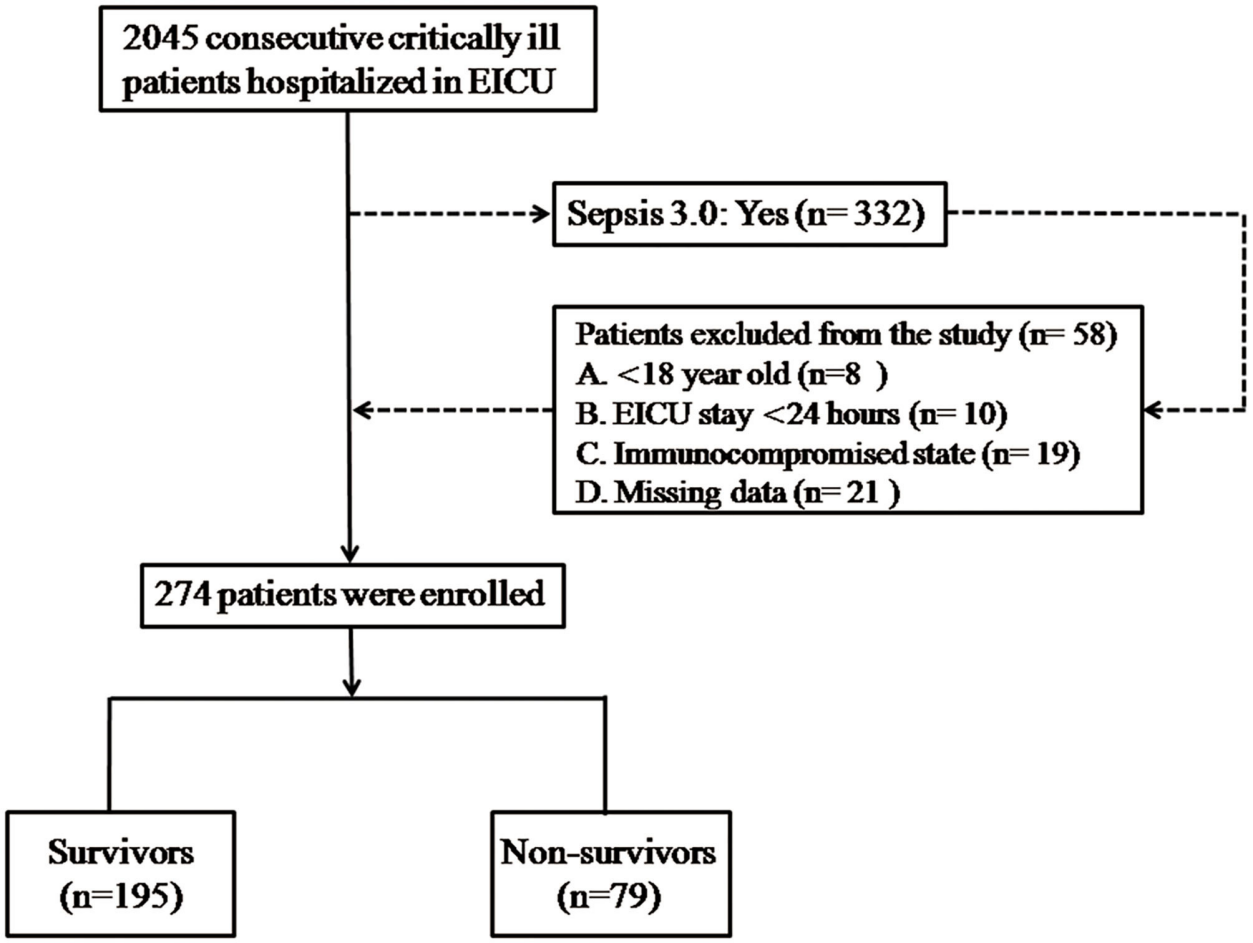
Flowchart of the enrolled patients. There were 2,045 adult patients who met the diagnostic criteria of the Surviving Sepsis Guideline (Sepsis 3.0), who were tested in this study, and 264 enrolled patients were stratified into the survivor and non-survivor groups. Demographic data and clinical and laboratory parameters were compared between the survivor and non-survivor groups.

This single-center retrospective observational study was administrated in septic patients who were admitted to the emergency ICU (EICU) of the Second Hospital of Hebei Medical University between January 2015 and December 2020; 2045 adult patients (aged >18 years) who conformed to the diagnostic standard of the Surviving Sepsis Guideline (Sepsis 3.0) (12) were enrolled in this study. We excluded patients with a reference standard that included (1) younger than 18 years old, (2) EICU hospitalized <24 h, (3) immunodeficient state: systemically solid tumor or hematological malignances [active stage or decubation within 5 years; recipients of autotransplantation or allotransplantation in stem cell; solid organ transplantation, long-term application for hormonotherapy (>30 days) or high dose (>1 mg/kg/day) of steroids (>14 days)] or currently on immunosuppressive drug for more than 30 days, (4) acute or chronic liver disease, (5) using antihyperlipidemic therapy, (6) synsemantic or missing medical data registers. The enrolled participants signed informed consent forms and were observed for at least 28 days. The patients accepted professional medical care in the whole hospitalized course and normative treatment complying with the Surviving Sepsis Campaign Guideline (12). The research was conducted in accordance with the principle of the Declaration of Helsinki and approved by the Medical Ethics Committee established in the Second Hospital of Hebei Medical University, Shijiazhuang, China (Figure 1).

### Data Extraction Process

Demographic data and clinical and laboratory parameters of enrolled patients were collected from the electronic medical record system in the hospital. Patients' demographic characteristics are documented in detail in the records, and complications; source of infection; and vital signs, including body temperature, systolic blood pressure (SBP), and heart rate are collected. Laboratory parameters were obtained from the examination of blood samples in the antecubital vein within 3 h after admission to the EICU. Routine hemogram was determined with the EB-10 (F4) mechanized hematology analytical facility (Sysmetix, Mobe, Japan), and procalcitonin (PCT) level was tested by the luminescence immunoassay instrument (Goche, socobas e211). Electromagnetic biochemical analysis equipment 730-128 (Mitachi High Technologies, Japan) was used to determine the biochemical parameters. C-reactive protein (CRP) level was measured by applying the CRP mensurable device (Quickly Read system). In addition, MHR was obtained by calculating a ratio of absolute monocyte count to high-density lipoprotein (HDL) cholesterol content. NLR was computed by distributing the neutrophil-to-lymphocyte count. Of note, each participant is evaluated as to severity degree by means of Sequential Organ Failure Assessment (SOFA) and Acute Physiology and Chronic Health Evaluation II (APACHE II) scores. We employed the all-cause mortality of hospitalization as a primary endpoint in the current study.

### Statistical Analysis

All statistical processes were performed by using SPSS software (version 26.0; IBM Corporation, St. Louis, Missouri, USA). Enrolled patients, who were stratified into survivor and non-survivor groups in line with 28-day survival status, are compared by the baseline characteristics accordingly. Continuous data that conformed to normal distribution were expressed as mean ± standard deviation (SD), non-normally distributed variables were presented as median (interquartile range), and categorical data were shown as counts (percentages). We adopted Student's *t*-test or the Mann–Whitney *U* test to evaluate the differences between continuous variables in the two groups, and the chi-squared test was applied to the comparison of categorical variables.

Prior to the analysis of regression on model risk factors, we selected boxplots to demonstrate the correlation between normal numerical variables (CRP, PCT, NLR, MHR) and severity of sepsis. Univariate and multivariate analyses were conducted by Cox regression model to assess the predicted potential of the abovementioned markers on 28-day mortality in sepsis. All the variables in the Cox regression models are shown as hazard ratio (HR) within 95% confidence intervals (CIs). Univariate analysis was performed preferentially, and variables with significance determined as *P* < 0.01 should subsequently be incorporated into the multivariate model, which is adjusted for the factors of age, sex, BMI, SBP, APACHE II, and SOFA scores. In a multiple Cox regression model, we compare the predictive value of PCT, CRP, NLR, and MHR.

Before further assessing the accuracy of the parameters in predicting the prognostic value of sepsis with the measure of AUC in the ROC analysis (25) and classification of mortality risk categories in the following step (26, 27), we adopted logistic regression to calculate and output a proportion of combination between NLR and MHR, which constructed a new model defined as NLR_MHR. The predictive value of each model of CRP, PCT, NLR, MHR, and NLR_MHR was evaluated by ROC analysis. Additionally, the sensitivity and specificity of the optimal cutoff value, positive predictive value (PPV) and negative predictive value (NPV) as well as Youden index were calculated to evaluate the accuracy of the predictors. The effects of classified model mortality risk categories were analyzed with net reclassification improvement index (NRI), which represents the reformative ability in differentiation and reclassification as well as the integrated discrimination improvement index (IDI) that was appropriate for the new model in prediction (27). The values of NRI and IDI were calculated by R Statistical Software (version 4.0.3, Vienna, Austria). *P* value <0.05 was considered statistically significant.

## Results

### Baseline Characteristics and Clinical Outcomes

A total of 274 eligible patients admitted to the EICU in the period of study were enrolled with reference to the detailed flow diagram shown in Figure 1 and should be classified into survivor (*n* = 195) and non-survivor groups (*n* = 79), according to the eventual state. The difference in demographic data of groups and comparative results are represented in Table 1. Of all septic patients, 195 (71.2%) patients survived more than 28 days. In comparison to those in the survivor group, patients in the non-survivor group, respectively, presented older ages (59.47 ± 7.44 vs. 56.96 ± 11.67; *P* < 0.05), higher body temperatures (38.58 ± 0.89 vs. 38.17 ± 0.88; *P* < 0.05), and lower levels of SBP (107.22 ± 15.97 vs. 112.35 ± 14.90; *P* < 0.05). Meanwhile, patients of the non-survivor group were more likely to be complicated with hypertension and coronary heart disease than those of the survivor group. With regard to the laboratory data, the levels of neutrophils (6.69 ± 1.42 vs. 6.12 ± 1.26) and monocytes (0.67 ± 0.66 vs. 0.41 ± 0.07) in the non-survivor group were significantly higher, whereas the levels of lymphocyte (1.12 ± 0.39 vs. 1.57 ± 0.65) and HDL (46.53 ± 2.4 vs. 52.11 ± 2.16) were markedly lower than those of the survivor group. For bioindicators, the levels of PCT (10.39 ± 4.21 vs. 7.71 ± 3.63; *P* = 0.001), NLR (6.28 ± 1.37 vs. 4.28 ± 1.18; *P* = 0.001), and MHR (14.29 ± 2.52 vs. 9.76 ± 2.85; *P* = 0.001) in the non-survivor group were markedly elevated than those in the survivor group. Otherwise, there was no significant difference of CRP level between the survivor and non-survivor groups. Apparently, patients in the non-survivor group presented poorer prognoses with the outcomes of APACHE II scores (27.11 ± 3.86 vs. 17.05 ± 4.13; *P* = 0.001) and SOFA scores (12 (10–14) vs. 7 (6–9); *P* = 0.001). Nevertheless, we failed to observe significant differences in terms of BMI, heart rate, and source of infection.

**Table 1:** Baseline characteristics of studied population.

**Variables**	**Total**	**Survivors**	**Non-survivors**	***P***
	***n*** **= 274**	***n*** **= 195**	***n*** **= 79**	
**Demographics**				
Sex (male %)	168 (61.3)	117 (60.0)	51 (64.6)	0.483
Age, years	57.68 ± 10.65	56.96 ± 11.67	59.47 ± 7.44	0.035
BMI, kg/m^2^	22.84 ± 2.84	22.68 ± 2.73	23.22 ± 3.09	0.148
Body temperature, °C	38.29 ± 0.90	38.17 ± 0.88	38.58 ± 0.89	0.011
SBP, mmHg	110.87 ± 15.34	112.35 ± 14.90	107.22 ± 15.97	0.012
Heart rate, bpm	98.07 ± 17.36	97.27 ± 17.23	100.04 ± 17.74	0.234
**Site of primary infection**				
Lower respiratory tract	101 (36.9)	68 (34.9)	33 (41.8)	0.350
Intra-abdomen	64 (23.4)	45 (23.1)	19 (24.1)	
Urinary system	45 (16.4)	37 (18.9)	8 (10.1)	
Skin and soft tissue	45 (16.4)	30 (15.4)	15 (18.9)	
Unknown origin	19 (6.9)	15 (7.7)	4 (5.1)	
**Comorbidities**				
Hypertension	80 (29.2)	49 (25.1)	31 (39.2)	0.020
CHD	45 (16.4)	17 (8.7)	28 (35.4)	0.001
Diabetes mellitus	53 (19.3)	32 (16.4)	21 (26.6)	0.053
COPD	19 (6.9)	11 (5.6)	8 (10.1)	0.186
Cerebrovascular disease	45 (16.4)	27 (13.8)	18 (22.8)	0.070
CRI	29 (10.6)	17 (8.7)	12 (15.2)	0.115
Malignant neoplasm	13 (4.7)	8 (4.1)	5 (6.3)	0.432
**Laboratory data**				
CRP, mg/L	88.84 ± 4.39	88.55 ± 3.99	89.57 ± 5.22	0.082
PCT, ng/ml	8.48 ± 3.98	7.71 ± 3.63	10.39 ± 4.21	0.001
Neutrophil, *10^9^/L	6.28 ± 1.33	6.12 ± 1.26	6.69 ± 1.42	0.002
Lymphocyte, *10^9^/L	1.44 ± 0.62	1.57 ± 0.65	1.12 ± 0.39	0.011
Monocyte, *10^9^/L	0.48 ± 0.14	0.41 ± 0.07	0.67 ± 0.66	0.001
HDL, mg/dl	49.13 ± 2.31	52.11 ± 2.16	46.53 ± 2.4	0.015
NLR	4.86 ± 1.53	4.28 ± 1.18	6.28 ± 1.37	0.001
MHR	11.07 ± 3.43	9.76 ± 2.85	14.29 ± 2.52	0.001
Severity scores				
APACHE II	19.95 ± 6.09	17.05 ± 4.13	27.11 ± 3.86	0.001
SOFA	8 (7–10)	7 (6–9)	12 (10–14)	0.001

*Data were expressed as mean ± standard deviation (SD), median (interquartile range) or No. (%). P < 0.05 indicates statistical significance.*

*CHD, coronary heart disease; COPD, chronic obstructive pulmonary disease; APACHE II, Acute Physiology and Chronic Health Evaluation II; SOFA, Sequential Organ Failure Assessment*.

### Correlation Analysis of Investigated Variables and Severity Scores

**Figure 2:**
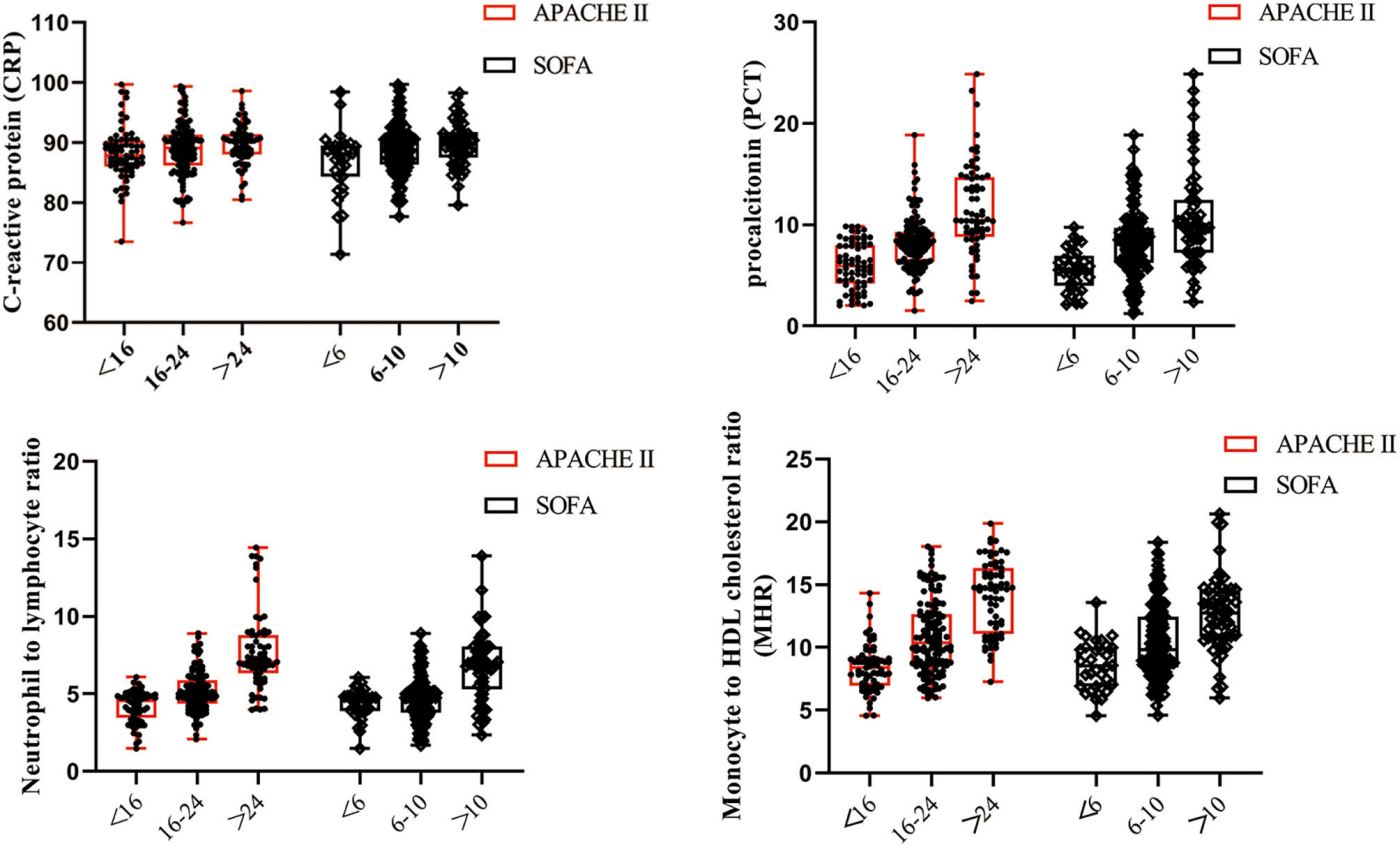
Levels of CRP, PCT, NLR, and MHR in patients with severity classifications. Scatterplots were conducted to demonstrate the correlation between laboratory bioindicators and severity of sepsis, which was evaluated by APACHE II and SOFA scores. MHR and NLR were more closely relevant to the severity of sepsis than CRP and PCT.

Scatterplots were distributed to describe the correlation between laboratory bioindicators and severity of sepsis. In antecedent of the statistical analysis, we stratified septic patients into three groups complying with the following levels of scores: (1) APACHE II scores: <16, 16–24, >24 and (2) SOFA scores: <6, 6–10, >10 (28, 29). As shown in Figure 2, MHR presented the closest correlation with both APACHE II and SOFA scores, followed by NLR and PCT, whereas CRP showed absence of relevance to severity scores.

### Prognosticators of 28-Day Mortality Risk in Septic Patients

To identify the risk factors for 28-day mortality in patients suffering from sepsis, we implemented Cox regression analysis. As revealed in Table 2, high levels of PCT, NLR, and MHR potentially aggravated the 28-day mortality risk of septic patients (*P* < 0.001). In multivariate analyses, it indicated that MHR remained statistically significant after adjusting for age, sex, BMI, SBP, and APACHE II as well as SOFA scores (HR = 1.217, 95% CI 1.112–1.331, *P* < 0.001). Additionally, NLR was confirmed to be independently associated with 28-day mortality of patients diagnosed with sepsis (HR = 1.404, 95% CI 1.170–1.684, *P* < 0.001).

**Table 2:** Hazard ratio of predictors in univariate and multivariate Cox regression.

**Variables**	**Univariate**		**Multivariate**	
	**HR (95% CI)**	***P***	**HR (95% CI)**	***P***
CRP	1.047 (0.992–1.106)	0.097	1.004 (0.947–1.064)	0.900
PCT	1.096 (1.038–1.158)	0.001	1.024 (0.954–1.098)	0.517
NLR	1.960 (1.703–2.256)	<0.001	1.404 (1.170–1.684)	<0.001
MHR	1.410 (1.307–1.520)	<0.001	1.217 (1.112–1.331)	<0.001

*The multivariate model included age, sex, BMI, SBP, APACHE II, and SOFA scores. P < 0.05 indicated statistical significance.*

*HR, hazard ratio; NLR, neutrophil-to-lymphocyte ratio; CRP, C-reactive protein; PCT, procalcitonin; MHR, monocyte/HDL cholesterol ratio*.

### The Predictive Accuracy of Parameters for 28-Day Mortality in Septic Patients

**Figure 3:**
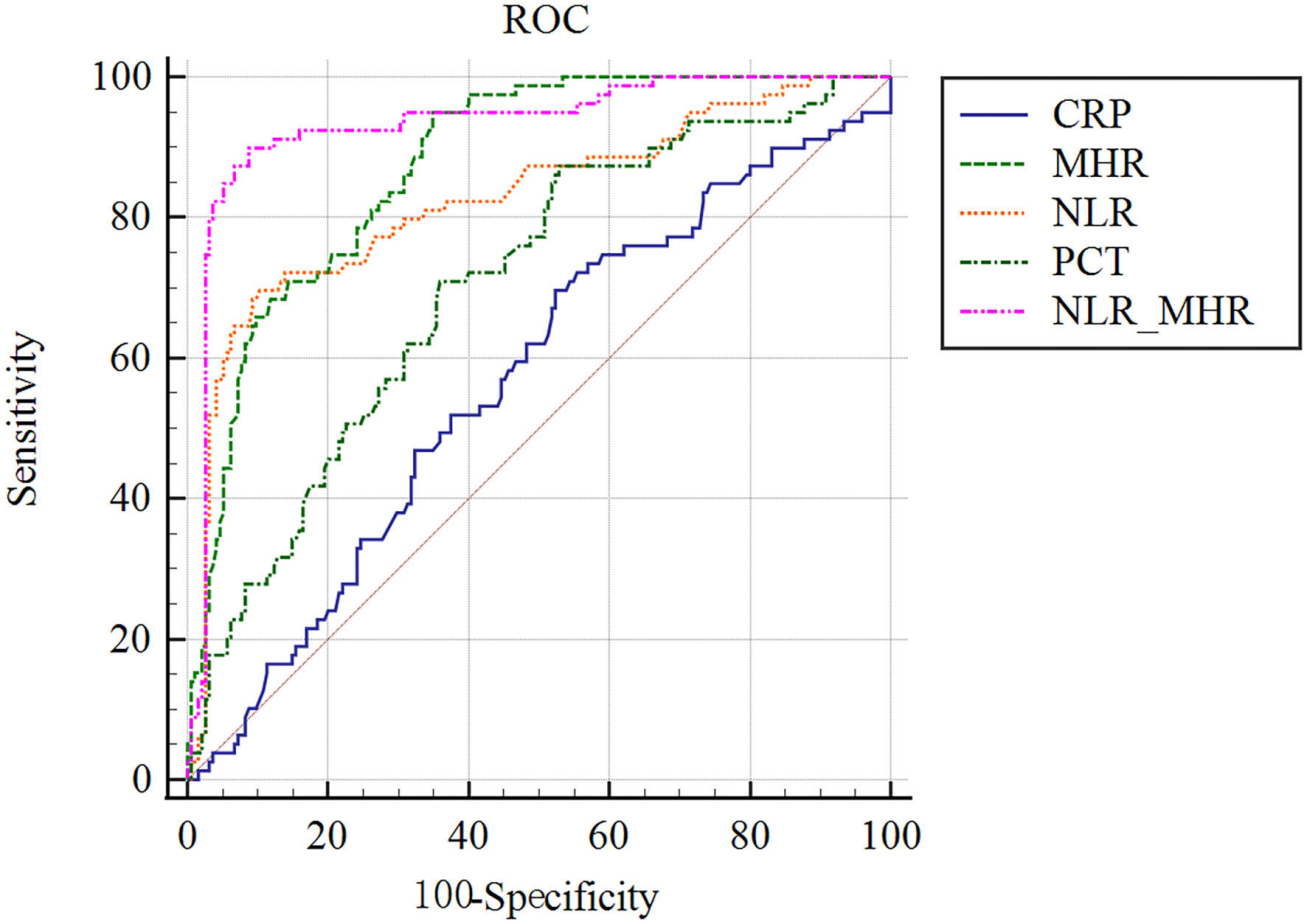
ROC analysis of parameters for predicting the prognosis of 28-day mortality in sepsis. MHR_NLR model displayed the largest AUC than other indicators. The AUC value of CRP was found to be the minimum, which were talentless in predicting sepsis prognostic risk.

We compared the model performance–discrimination, overall fit, and reclassification to further evaluate the predictive potency of 28-day mortality in sepsis. For the model discrimination displayed in Figure 3, outcomes in the AUC diagram indicate that the MHR_NLR model had the largest AUC (0.934 [0.898–0.960]), followed by MHR (0.876 [0.831–0.913]), NLR (0.827 [0.777–0.870]), and PCT (0.705 [0.647–0.758]). The AUC value of CRP (0.569 [0.508–0.628]) was found to be smaller than the above predictors, which demonstrate the serum CRP levels might be irrelevant to the mortality risk of septic complications.

Next, the model of overall reclassification improvement was assessed by NRI and IDI, which are more sensitive tests for improving model discrimination than ROC curves. When compared with CRP, it is noteworthy that PCT failed to indicate the significantly higher NRI and IDI. NLR shows a better NRI compared with PCT. Additionally, MHR with high IDI and NRI could better regrade patients to a more proper mortality risk classification than CRP, PCT, and NLR. Notably, we identified that NLR_MRH could better reclassify patients as indicated by significantly higher NRI and IDI in comparison to the single of NLR or MHR (Table 3).

**Table 3:** Evaluating the efficiency of parameters in improving to predict 28-day death risk of sepsis.

**Variables**	**CRP**	**PCT**	**NLR**	**MHR**	**NLR_MHR**
AUC	0.569	0.705	0.827	0.876	0.934
	(0.508 to 0.628)	(0.647 to 0.758)	(0.777 to 0.870)	(0.831 to 0.913)	(0.898 to 0.960)
	NA	*P* < 0.001	*P* < 0.001	*P* < 0.001	*P* < 0.001
IDI	NA	0.054	0.062	0.155	0.185
		−0.074 to 0.264	−0.028 to 0.159	0.012 to 0.326	0.019 to 0.236
		*P* = 0.236	*P* = 0.127	*P* = 0.03	*P* = 0.02
NRI	NA	0.062	0.251	0.351	0.378
		0.0068 to 0.096	0.0098 to 0.435	0.116 to 0.549	0.096 to 0.576
		*P* = 0.248	*P* = 0.04	*P* < 0.001	*P* = 0.023

*Pairwise statistical comparisons were conducted from left to right. P < 0.05 indicated statistically significant.*

*AUC, area under the ROC curve; IDI, integrated discrimination improvement; NRI, net reclassification improvement*.

### ROC Curve Analysis

To achieve more precise progress in predictive value for 28-day mortality of sepsis, we figured out the sensitivity, specificity, cutoff point, PPV, NPV, and Youden index in ROC analysis. As shown in Table 4, the sensitivity and specificity of MHR at the optimal cutoff value of 10.15 were 94.94% (87.5–98.6%) and 65.13% (58.0–71.8%), respectively. The optimal cutoff value of NLR was 5.51, which gave a sensitivity of 69.62% (58.2–79.5%) and a specificity of 89.74% (84.6–93.6%). Of note, MHR_NLR trended toward a high specificity of 91.28% (86.4–94.8%). Based on the cutoff values listed in Table 4, NLR_MHR obtained a best PPV (80.7, 72.5–86.9%), and MHR showed a best NPV (96.9, 92.4–98.8%). Furthermore, we found NLR_MHR presented the highest Youden index of 0.8116. Collectively, NLR_MHR was determined to be the most reliable diagnostic accuracy for predictive value of 28-day mortality in septic patients.

**Table 4:** Diagnostic value of the predictive parameters.

**Variables**	**Sensitivity**	**Specificity**	**Cut-off point**	**PPV**	**NPV**	**Youden index**
	**(95% CI)**	**(%)**		**(95% CI)**	**(95% CI)**	
CRP	69.62	47.69	88.4	35	79.5	0.1731
	58.2–79.5	40.5–54.9		30.7–39.7	72.9–84.8	
PCT	70.89	64.10	8.51	44.4	84.5	0.3499
	59.6–80.6	56.9–70.8		38.7–50.3	79.1–88.6	
NLR	69.62	89.74	5.51	73.3	87.9	0.5936
	58.2–79.5	84.6–93.6		63.9–81.0	83.9–91.1	
MHR	94.94	65.13	10.15	52.4	96.9	0.6006
	87.5–98.6	58.0–71.8		47.5–57.4	92.4–98.8	
NLR_MHR	89.87	91.28	NLR = 5.08	80.7	95.7	0.8116
	81.0–95.5	86.4–94.8	MHR = 13.47	72.5–86.9	92.0–97.7	

## Discussion

Sepsis has recently been redefined as a syndrome of physiological, pathological, and biochemical abnormalities that induce an uncontrollable host reaction to inflammation that causes fatal multiple organ dysfunction (1, 12). A vicious circle of inflammation and oxidative stress ultimately inducing immunosuppression is supposed to be the essence of the pathophysiological process. The incidence of sepsis has been gradually trending toward ascension, and conservative estimates indicate that sepsis may be a leading cause of death in ICU hospitalization (30, 31). Therefore, early identification of septic risk is critical to improving the diagnosis, therapeutic intervention, and prognosis in this serious complication. Although a variety of predictive models for sepsis risk factors are established in clinical studies, the practical values of these biomarkers are still disputed. Accordingly, the current study was conducted to explore a novel death risk screening indicator defined as MHR combined with NLR in predicting 28-day mortality in sepsis, and further evaluate the predictive efficacy of the parameters.

It is demonstrated that sepsis is induced by a dysregulated host response to infection, and the innate immune system is triggered when microbial molecular patterns are identified by specific receptors expressed on immune cells (32). This interaction can activate immune cells, including neutrophils and lymphocytes, to release both proinflammatory and anti-inflammatory mediators. Neutrophils as the first line of defense play an indispensable role in elimination of pathogens by phagocytosis and T cell activation, and lymphocytes as an indicator of immunosuppression play a role in mediating apoptosis (33, 34). Therefore, NLR appears to represent a balanced state between innate and adaptive immunity. Recently, several reports have documented the potential utility of NLR as a diagnostic parameter that is closely related to miscellaneous diseases, including inflammation, ischemic cerebrovascular disease, cancer, and trauma (20, 35–40). In our observational study, we found that NLR in patients who died were remarkably elevated compared with those with mild cases (*P* < 0.01) (41), which is in accordance with the previous study showing that an inflammatory reactive state typically led to neutrophilia and relative lymphocytopenia (42). The AUC value of NLR corresponded to 0.827 with 69.62% sensitivity and 89.74% specificity at the optimal cutoff value of 5.51. Theoretically, increased neutrophils reflect a response to microbial infection and migration to the infected region, whereas a reduced number in apoptotic lymphopenia contributes to the development of immunosuppression (43). A high level of neutrophils in the circulation in patients with sepsis indicate overactivation of the innate immune response (44). Conversely, exhaustion of lymphopenia may induce inefficiency in initiating an adaptive immune response and activating T cells. Hence, the results in our study verify the inference that NLR could be a prognostic sign of impending sepsis and predicting mortality risk in septicemia.

Oxidative stress is considered to be another crucial element involved in progression of sepsis. Increasing evidence suggests that both the incapacity of cells to consume oxygen and accumulation of peroxide may severely exacerbate the pathological process of sepsis (45). Sepsis-induced multiple organ dysfunctions finally occur when it suffers from an imbalance of oxidants and antioxidants due to capillary penetrability damage, deteriorative organic property, and hyporespiratory function induced by mitochondrial malfunction (46). During the process of oxidative stress, monocytes, the main source of proinflammatory and oxidative mediators, reveal the responsive capacity of the innate immune system (46). Although HDL cholesterol (HDL-C) inhibits hyperoxidation of low-density lipoprotein cholesterol (LDL-C) and can exert a protective effect on the endothelium. In the early stage of sepsis, circulating monocytes migrate to vascular endothelium and mature into macrophages, which then oxidize liposomes and differentiate into foam cells to release inflammatory cytokines and activate T lyphocytes and more monocytes. In contrast, HDL-C inhibits the activation and transformation of monocytes, thereby resulting in a suppression of inflammatory response (47, 48). Based on this view, it is rational to unite these two parameters into a single index (MHR), which is cost-effective and consists of easily available laboratory parameters reflecting anti-inflammatory and antioxidant effects. Recent studies propose a high level of MHR as an unfavorable prognostic marker, indicating systemic inflammatory and oxidative diseases, including Behçet's disease, psoriasis, and spondylarthritis (49–51). The present study was consistent with the abovementioned clinical observations in that the MHR level was noticed to be significantly higher in the non-survivors group with the average level than the survivor group, which demonstrated MHR was a more precise parameter than others for evaluating systematic inflammation in sepsis. Kanbay and colleagues analyzed the level of MHR in critical patients on admission, and they suggest that MHR might be an early predictor in cardiovascular emergency in patients with chronic kidney disease (24). Similar to previous reports, the scatter diagram in our results found that a high level of MHR was significantly correlated with APACHE II and SOFA scores. The fatality in patients with a high level of MHR was more serious than in patients with a low level of MHR, which indicates that MHR was independently related to prognosis risk for a septic event. Moreover, the current data supports previous findings that MHR is better than NLR in determining prognosis due to its larger AUC. When the cutoff value of MHR was set at 10.15, a higher sensitivity was obtained, and correspondingly, 52.4% of dead patients were effectively classified in the non-survivor group, and 3.1% of the deceased could be assigned to the surviving patients group. Strikingly, the addition of MHR could efficaciously improve the early diagnosis capacity according to the high IDI and NRI. These findings illustrate that MHR may be advantageous in diagnosing sepsis and predicting prognostic risk of 28-day mortality.

Other markers, including CRP and PCT, were also analyzed in our study, and their predictive abilities appeared negative, and they failed to forecast progressive bacterial infection in terms of sepsis in early diagnostic settings. Cox analysis indicated that PCT was incapable of assessing septic mortality after adjusting partial factors (HR = 1.024, 95% CI 0.954–1.098, *P* = 0.517). Clinically, due to the trend of PCT reaching a plateau slowly at 8–24 h, the evaluated capacity of PCT in predicting septic prognosis was obviously disadvantaged with an AUC of 0.705, smaller than those of NLR and MHR. Nevertheless, CRP released in the acute phase of inflammation indicated no difference between the survivor and non-survivor groups and were irrelevant to the severity of sepsis. The potential cause might be that the CRP level peaked within only 48 h and failed to reflect the terminal state of sepsis (52).

Because MHR obtained higher sensitivity but lower specificity than NLR, we conducted model parameters of MHR together with NLR in predictive risk of sepsis, and further evaluated the efficiency in predicting 28-day mortality. In the predictive model of MHR_NLR, the AUC maximum value was 0.934 with a better sensitivity and specificity than the single variable. Moreover, we examined such parameter in terms of reclassifying improvement and discrimination by use of IDI and NRI, which showed significant improvement in the mean difference of predicted probabilities. Taken together, MHR combined with NLR as the parameter is not only appropriate for the early diagnosis of sepsis, but also for the prediction of its severity and prognosis.

There are many clinical implements in the original study. We conducted multiple indicators to evaluate their predictive efficacy to ensure the reliability and accuracy. It first illuminated the inchoate predictive value of MHR combined with NLR for prognosis in sepsis, which provided a more precise guideline for administration and management of septic patients as well as clinical follow-up during the late stage of development. Continuous monitoring of these laboratory variables contributes to enhancing septic prognosis and treatment. Nevertheless, several limitations still remain in our current study. First, this single retrospective study needs to be further proven by more prospective cohort studies or multicenter randomized clinical trials. Futhermore, we need to assess the possible impact of patient's characteristics, including dietary and smoking history, which can affect performance evaluation. Second, a larger sample size appears to be essential to reduce proportional error. Finally, we should collect more information about mechanical ventilation and hemodynamic-associated indicators to better reflect the eventual development of sepsis.

## Conclusions

In summary, the current study suggests that MHR together with NLR are closely related to the severity of sepsis and might be independent predictors of 28-day mortality of septic patients. Notably, MHR combined with NLR can significantly improve the predictive efficiency of 28-day mortality in sepsis.

## Data Availability Statement

The original contributions presented in the study are included in the article/Supplementary Material, further inquiries can be directed to the corresponding authors.

## Ethics Statement

The studies involving human participants were reviewed and approved by Medical Ethic Committee of Second Hospital of Hebei Medical University, Shijiazhuang, China. The patients/participants provided their written informed consent to participate in this study.

## Author Contributions

J-yL performed the major design and drafted the manuscript. R-qY and S-qL conducted the statistics. Y-fZ contributed to the data collection. Y-pT and Y-mY conceptualized, supervised, and revised. All authors read and approved the final manuscript.

## Funding

This work was supported by grants from the National Natural Science Foundation of China (81730057, 81873946) and the Provincial Key Project of Medical Science Research of Hebei (20210013).

## Conflict of Interest

The authors declare that the research was conducted in the absence of any commercial or financial relationships that could be construed as a potential conflict of interest.

## Publisher's Note

All claims expressed in this article are solely those of the authors and do not necessarily represent those of their affiliated organizations, or those of the publisher, the editors and the reviewers. Any product that may be evaluated in this article, or claim that may be made by its manufacturer, is not guaranteed or endorsed by the publisher.
